# Can we quantify a safe margin to reduce local recurrence in parosteal osteosarcomas around the distal femur? A cohort study based on 27 patients

**DOI:** 10.2340/17453674.2026.45943

**Published:** 2026-05-22

**Authors:** Kumaran RASAPPAN, Giuseppe Francesco PAPALIA, Scott EVANS, Micheal PARRY, Jonathan STEVENSON, Guy MORRIS, Lee JEYS, Vineet KURISUNKAL

**Affiliations:** 1 Department of Oncology and Complex Arthroplasty, Royal Orthopaedic Hospital (ROH), Birmingham, UK; 2 Department of Orthopaedic Surgery, National University Hospital, Singapore; 3 Campus Bio-Medico University of Rome, Italy

## Abstract

**Background and purpose:**

Parosteal osteosarcoma of the distal femur usually presents as a posteriorly outgrowing tumor that lies close to the popliteal neurovascular bundle, making resection with a wide margin difficult. Tumors are often lower grade meaning surgery often forms the mainstay of treatment. We aimed to evaluate resection margins on local recurrence (LR) and metastasis-free survival rates of patients undergoing surgery for parosteal osteosarcoma of the distal femur.

**Methods:**

This retrospective study evaluated patients with histologically confirmed parosteal osteosarcoma of the distal femur at a single institution between 2000 and 2022, with at least 2 years of follow-up. Patients undergoing primary amputation, those with distant metastases at diagnosis, and those with insufficient clinical information were excluded from the study. Data such as postoperative margins, tumor grade, LR, and metastasis was collected and analyzed.

**Results:**

27 patients were included. Involved margin at resection was recorded in 4 patients: a closest margin of < 1 mm in 14 and ≥ 1 mm in 9. Local recurrence (LR) occurred in 8 patients. Compared with ≥ 1 mm margins, the risk of LR was higher for involved margins (risk difference [RD] 0.75, 95% confidence interval [CI] 0.21–0.95; relative risk [RR] 14.0, CI 0.88–222) and for < 1 mm margins (RD 0.36, CI 0.00–0.61; RR 7.33, CI 0.45–119). Metastasis-free survival showed similar trends. Compared with ≥ 1 mm margins, the risk of metastasis was higher for involved margins (RD 0.25, CI −0.11 to 0.70; RR 6.0, CI 0.29–122) and for < 1 mm margins (RD 0.36, CI 0.00–0.61; RR 7.33, CI 0.45–119). There was no significant difference between the tumor grade and rate of LR.

**Conclusion:**

A margin greater than or equal to 1 mm showed reduced LR and less metastasis in patients with parosteal osteosarcoma of the distal femur.

Parosteal osteosarcomas (POS) are a rare and distinct subtype of osteogenic malignant sarcoma that arise from periosteum [1]. They constitute about 4–6% of all osteosarcomas and arise most commonly in the femur (61.5%), humerus (15.9%), and tibia (12.8%) [2,3]. These tumors are characterized by their slow-growing nature, locally aggressive behavior, and relatively low metastatic potential compared with other osteosarcoma variants, as the majority are low-grade at presentation. However, intermediate and dedifferentiated high-grades are also seen, on occasion in association with a predominant low-grade tumor [4].

As with the majority of primary sarcomas of bone, local recurrence (LR) is associated with the margin achieved at resection, with a lower rate of recurrence seen when POS is excised with a wide margin [2,4-6]. LR has a significant negative effect on survival, as 80% of the local recurrences are at a higher grade than the primary tumor, or dedifferentiated, with an associated increased incidence of metastases [2]. The predominant low-grade nature of POS often renders neo- or adjuvant chemotherapy unpredictable [7,8] and thus the role of chemotherapy in the treatment of primary POS is not as evident as it is in the treatment of conventional osteosarcoma. As such, the mainstay for the treatment of POS remains excision with an adequate margin [7,8].

Because the majority of POS present as outgrowing tumors on the dorsal aspect of the distal femur [2], managing them with a wide surgical margin is often challenging, as the tumor often lies in close proximity to or abutting the popliteal neurovascular bundle (NVB). There is often a clinical dilemma as to whether surgeons should attempt limb salvage in POS around the distal femur, as achieving an adequate margin around the vascular bundle can be difficult to achieve. Inadequate margins are associated with a higher incidence of LR, which is often of a higher grade, and possible distant metastatic disease [2].

What still remains open to debate is what constitutes an adequate margin. Much effort has gone into defining the adequate margin to minimize LR in conventional osteosarcoma [10] but due to its relative rarity, its predominant resistance to chemotherapy, and the location of the extraosseous component of the disease, most frequently posterior to the femur and abutting the neurovascular bundle, the discussion regarding limb salvage vs limb sacrifice is often more pertinent in the case of POS when compared with conventional osteosarcoma. In many cases of limb salvage in POS of the distal femur, the posterior margin of the tumor is invariably the closest margin regardless of the extension of the tumor within bone.

We aimed to evaluate resection margins on LR and metastasis-free survival rates of patients with POS of the distal femur who were undergoing tumor resection surgery. We hypothesized that LR and the propensity for distant metastases in these tumors remain low despite close but clear margins.

## Methods

### Study design

We performed a single-center, retrospective study that included all patients with POS of the distal femur who underwent limb salvage surgery at a single bone sarcoma tertiary referral center in the United Kingdom with a catchment area of about 22 million people, over a 22-year period from 2000 to 2022. Patients were selected from a prospectively maintained sarcoma database at the hospital, which had received approval from the local ethics committee for patient data to be collected and stored. Selection bias was minimized by including all consecutive eligible patients from this database.

### Histology

All patients underwent biopsy of the lesion prior to surgery. In all cases, the management plan was discussed in a specialist sarcoma multidisciplinary team (MDT) meeting, including specialist pathologists, radiologists, and surgeons. In all cases, the decision regarding local control was based on the local and distant staging and the histological grade on biopsy. The decision for limb salvage in non-metastatic patients was based on an assessment of the projected margin that was expected to be achieved at resection, in particular, the proximity of the nearest vital structure, most often the popliteal vessels. In cases where an adequate margin was not anticipated, amputation was favored for local control.

### Inclusion and exclusion criteria

All patients who had a histologically confirmed diagnosis of POS arising from the distal femur were included in the study. Patients with a minimum 2-year period of follow-up were included. Patients undergoing primary amputation, those with distant metastases at diagnosis, and those with insufficient clinical information were excluded from the study.

### Data

Clinical and pathology data was collected by reviewing electronic records, and radiological data was collected from a picture archiving and communications system (PACS). The following data was collected from the patients eligible for the study: age, sex, tumor grade, postoperative resection margins in millimeters (mm), date of LR and/or metastasis, and time to final follow-up. Postoperative margins were classified into 3 categories: involved, < 1.0 mm, and ≥ 1.0 mm. Oncological outcomes, specifically, LR-free and metastasis-free survivals (MFS), were measured from the date of initial diagnosis to the detection of LR or metastatic disease or the last follow-up.

### Statistics

Statistical analysis was conducted using SPSS (version 26.0; IBM Corp, Armonk, NY, USA). Continuous variables were expressed with mean and standard deviation (SD); categorical variables were shown with frequency distribution and percentage. The Kaplan–Meier method and the log-rank test evaluated LR-free and metastasis-free survivals through univariate analysis. Hazard ratios (HR) and their 95% confidence intervals (CI) were calculated using the Cox regression model. Statistical significance was considered as P < 0.05 [9]. The study is reported according to STROBE guidelines.

### Ethics, data sharing plan, funding, use of AI, and disclosures

This research complies with the principles laid down in the World Medical Association Declaration of Helsinki. Patients were selected from a prospectively maintained sarcoma database at the hospital, which has received approval from the local ethics committee for patient data to be collected and stored.

No external funding or grants were received for any aspect of this work. The authors have not sought any writing assistance for the production of the paper. AI tools were not used. On behalf of all authors, the corresponding author states that there are no financial and personal conflicts of interest in this work. Complete disclosure of interest forms according to ICMJE are available on the article page, doi: 10.2340/17453674.2026.45943

## Results

**Figure 1:**
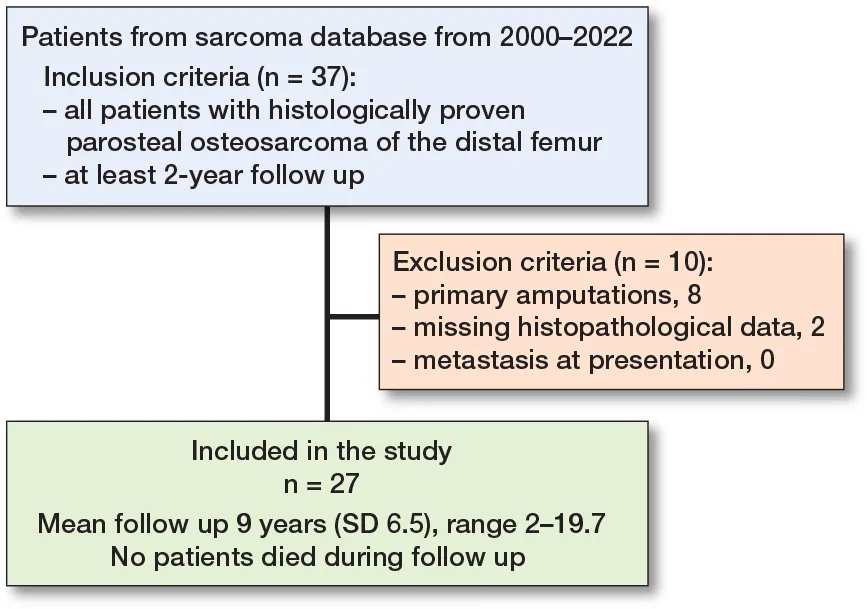
Patient flowchart.

37 patients with POS of the distal femur were identified during the study inclusion period. 8 patients underwent primary amputations, 2 patients had missing data on histopathological margins, and none of them had metastasis at presentation. After applying our inclusion and exclusion criteria, the study population comprised 27 patients with POS of the distal femur (Figure 1). No patients were excluded from the study due to metastasis at presentation as all POS were localized at presentation in our database. The mean age was 30 (SD 13) years, ranging from 11 to 58 years. There were 17 females and 10 males. All patients underwent local and distant staging prior to biopsy. In all patients, the biopsy tract was in a plane that could be resected at the time of definitive resection.

### Surgery

Surgery comprised an en-bloc or a shark-bite excision of the distal femur via an antero-medial approach or a lateral approach. In all cases, the biopsy tract was resected at the time of definitive resection, in continuity with the bone. Surgeon analysis of the resected specimen suggested a macroscopically clear margin in all cases. All resections comprised an intra-articular resection of the distal femur with reconstruction of the defect using a distal femoral endoprosthetic replacement except for 2 cases who underwent a shark-bite excision and reconstruction with structural bone graft.

All patients underwent sarcoma surveillance in keeping with nationally agreed standards, with clinical and radiological surveillance of the operated-on limb beyond 10 years. The mean follow-up period was 9 (SD 6.5) years, ranging from 2 to 19.7 years.

### Histology

Post resection pathology assessment comprised an assessment of histotype and grade, as well as macro- and microscopic assessment of resection margin at both the bone margin and the closest soft tissue margin. The margin status was involved in 4 resections, < 1 mm in 14, and ≥ 1.0 mm in 9 resections. Tumor grade was low grade in 11, intermediate grade in 10, high grade in 5 and dedifferentiated in 1. 16 did not undergo chemotherapy. 6 underwent neoadjuvant and 5 underwent adjuvant chemotherapy based on the presence of a higher grade component in the post resection specimen.

### Local recurrence

LR occurred in 8 out of 27 patients. In the < 1 mm margin group, 5 of 14 developed LR. The initial resection for those who developed recurrence was intralesional in 3 and < 1 mm in 5. No patients with a resection margin of ≥ 1 mm developed LR. Compared with ≥ 1 mm margins, LR risk was higher for involved margins (risk difference [RD] 0.75, CI 0.21–0.95; relative risk [RR] 14.0, CI 0.88–222) and for < 1 mm margins (RD 0.36, CI 0–0.61; RR 7.33, CI 0.45–119), with P = 0.02. Comparing involved margins with < 1 mm margins and LR, RD was 0.39 (CI −0.12 to 0.67) and RR 2.10 (CI 0.85–5.2). Of the 8 LRs, 3 were in low-grade tumors, 3 were in intermediate-grade tumors and 2 were in high-grade tumors. The incidence of LR in low-grade tumors was 3/11, 3/10 for intermediate grade, and 2/6 for high grade. The 1 patient with a dedifferentiated POS did not develop LR. There was no significant association between tumor grade and LR. Compared with low-grade tumors, LR risk for intermediate-grade tumors was RR 1.10 (CI 0.28–4.3) and RR 1.47 (CI 0.35–6.2) for high-grade tumors (P = 0.9). In relation to the location of the closest margin, in all cases where the margin was involved or < 1 mm, this closest margin was on the posterior aspect of the femur where the posterior soft tissue extension abutted the neurovascular bundle.

7 of the 8 cases with LR showed an increase in the tumor grade on histopathological analysis when the LR was resected as part of subsequent management. Of the 3 low-grade tumors, 1 remained low grade, 1 became an intermediate grade, and the other became a dedifferentiated tumor. Of the 3 intermediate-grade tumors, 2 became high grade and 1 became a dedifferentiated tumor. Both of the 2 high-grade tumors became dedifferentiated.

### Survival and metastases

Half the patients with LR developed metastatic disease. 3/4 of these patients had a dedifferentiated LR and 1 of them had a high-grade LR. However, 2 patients developed metastasis without LR. In both these patients, the tumor was a low grade at presentation.

**Figure 2:**
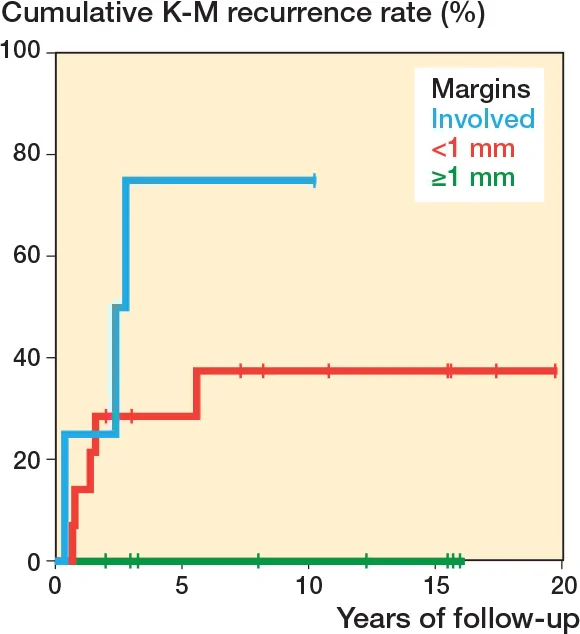
Kaplan–Meier (K–M) curve demonstrating cumulative local recurrence rates in patients with parosteal osteosarcomas of the distal femur.

LR-free survival at 5 years was affected by the development of LR. The LR-free survival at 5 years was 70% for the study population. The 5-year LR-free survival was 25% (CI 0.1–67) for tumors resected with involved margins, 64% (CI 34–84) for margins < 1 mm, and 100% for margins ≥ 1 mm. Patients with involved margins and margins < 1 mm were associated with significantly lower 5 year LR-free survival compared with those with ≥ 1 mm margins (log-rank P = 0.03) (Figure 2).

**Figure 3:**
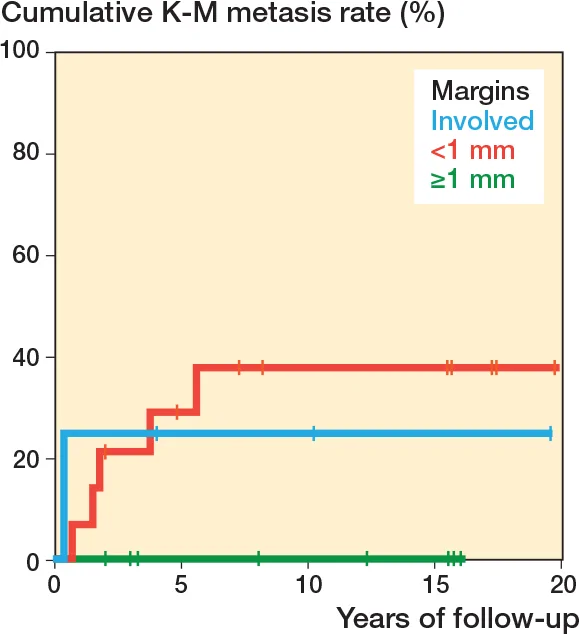
Kaplan–Meier (K–M) curve demonstrating cumulative metastasis rates in patients with parosteal osteosarcomas of the distal femur.

During the 22-year study period, 6 of 27 patients developed metastases. Metastases developed in 1 of 4 patients with involved margins, 5 of 14 with margins < 1 mm, and none of the 9 patients with margins ≥ 1 mm. Compared with ≥ 1 mm margins, metastasis risk was higher for involved margins (RD 0.25, CI −0.11 to 0.70; RR 6, CI 0.29–122) and for < 1 mm margins (RD 0.36, CI 0–0.61; RR 7.33, CI 0.45–119), with P = 0.1. Comparing involved margins with < 1 mm margins, the metastasis contrast was RD −0.11 (CI −0.43 to 0.38) and RR 0.7 (CI 0.11–4.4). The development of metastases was not significantly affected by the initial grade of the resected tumor. 3 of 11 patients with low-grade tumors developed metastases, 1 of 10 with intermediate-grade, and 2 of 5 with high-grade tumors developed metastases. The 1 patient with a dedifferentiated tumor did not develop metastases. Compared with low-grade tumors, the relative risk of metastasis was 0.37 (CI 0.05–2.6) for intermediate-grade tumors and 1.47 (CI 0.32–6.7) for high-grade tumors (P = 0.4). Overall, metastasis-free survival at 5 years was 78%. The 5-year metastasis-free survival rate was 75% (CI 13–96) for involved margins, 64% (CI 34–84) for margins < 1 mm, and 100% for margins ≥ 1 mm. Patients with involved margins and margins < 1 mm were associated with lower 5-year metastasis-free survival compared with those with ≥ 1 mm margins (log-rank P = 0.08) (Figure 3).

None of the patients included in the study died during the follow-up period.

## Discussion

We aimed to evaluate resection margins on LR and metastasis-free survival rates of patients with POS of the distal femur and showed that when resected with a margin of 1 mm or greater, the incidence of LR is significantly reduced when compared with a resection margin less than 1 mm, regardless of the grade of the resected tumor.

Although adherence of tumor mass to the NVB is associated with a higher rate of recurrence [11], some surgeons believe that the adventitia layer and the epineurium act as a thin barrier against tumor penetration into the NVB as suggested by Kawaguchi et al. [12] and opt for limb salvage surgery in POS. There has been a suggestion that the LR rate was less in a planned marginal excision around the NVB in POS than when it was done to shell out a presumptively benign lesion [11]. Jamshidi et al. studied the absence of a continuous rim of fatty tissue between POS of the distal femur and the posterior NVB, which did not increase the risk of LR in low-grade POS [13]. Although some of these studies suggested that a good qualitative margin was sufficient, none included a measurement of margin, making extrapolation difficult. Although the histological analysis of resected specimens did not report the quality of margins in our study, in contrast to these studies we have been able to quantify a minimum metric measurement above which the incidence of LR is significantly reduced.

Similar to other studies, our study showed that the majority of POS were low grade, with the minority of them being high grade or dedifferentiated tumors at presentation [2,3]. In contrast to other studies, however, we did not observe a correlation between grade and risk of LR, regardless of margin [3]. This may reflect the narrow inclusion criteria of our study, which aimed to look only at tumors of the distal femur, with a resultant small number of higher and dedifferentiated grades. Song et al. demonstrated a high correlation between margin and LR, with all patients undergoing intralesional resection suffering LR, 40% with marginal margins developing LR and none with wide margins developing LR [14]. However, based on these findings, the authors defined a wide margin as being more than 3 mm of normal soft tissue. In keeping with these findings, we were able to demonstrate a correlation between margin status and LR but were able to further reduce the definition of “adequate” to 1 mm of normal tissue.

Our data showed that in the 8 cases with LR, the median time to LR was 18 months (interquartile range 9–31). The time to LR in our study was in keeping with existing evidence [2,3,13]. As POS has been considered to be a low-grade, slow-growing tumor it would be expected that the time to LR would be longer. One postulation as to why LR seemed to be faster in POS was that the LRs were often associated with a higher grade tumors (including dedifferentiation) on recurrence. 7 of the 8 of our LR cases showed an increase in the tumor grade on histopathological analysis and this is also in keeping with existing literature [2]. This could account for the much more aggressive rate at which LR occurs as compared with other slow-growing low-grade tumors.

Although Ruengwanichayakun et al., who had analyzed 195 patients with POS from 1900 to 2018, showed that dedifferentiated POS had worse 5-year (65% vs 96%) and 10-year survival (60% vs 96%) survival compared with conventional POS [3], our paper failed to show any significance among 5-year metastasis-free survival between margins and tumor grade. This may be attributable to the low numbers in our study.

In our series, in patients with POS where LR did occur, 7 out of 8 turned out to be a higher grade than the original tumor that was resected. These findings were in keeping with the findings of Laitinen et al., where the author reported about 67% of low-grade tumors having a higher grade on LR [2]. Similar to Laitinen’s study, 50% of our patients with LR developed metastasis. In our series, all metastasis occurred in tumors with high grade or dedifferentiated LR. For these reasons, where an acceptable margin is debatable preoperatively, an amputation still needs to be considered, as LR often presents as higher grade tumors, which have an increased risk of distant metastatic disease and this has a significant negative effect on survival [2].

### Limitations

This is a retrospective study with only 27 patients due to the rarity of this particular subset of tumor. The quality of margins was not captured in our histopathology report, as many other studies have mentioned that a good-quality margin is better than a large but poor-quality margin. Patients undergoing primary amputation were excluded from our study (see Figure 1), and this could have affected our reporting of overall LR and metastasis-free survival in POS patients. However, this paper has evidence that in POS around the distal femur, close margins of ≥ 1mm can give good oncological outcomes, and primary amputations may not need to be considered in tumors that lie close to the popliteal NVB.

### Conclusion

We showed that a margin greater than or equal to 1 mm had reduced LR and less metastasis.

*In perspective*, obtaining wide margins in patients with POS of the distal femur remains challenging as the tumor is often in close proximity to the popliteal NVB. In light of our findings, achieving a margin ≥ 1 mm seems preferable for optimal oncological outcomes in POS around the distal femur.

## Notes

All authors contributed equally to the literature review, manuscript writing and review, and approved the version submitted for publication. Kumaran Rasappan (KR) is the corresponding author for this study and, as such, takes responsibility for the integrity of the data in the study and the accuracy of the content provided. KR had full access to all the data in the study and had the final responsibility for the decision to submit for publication.

Handling co-editors: Ivan Hvid and Robin Christensen

*Acta* thanks Thomas Baad-Hansen and Aare Märtson for help with peer review of this manuscript.
